# Integrating Textual Features with Survival Analysis for Predicting Employee Turnover

**DOI:** 10.3390/bs16020174

**Published:** 2026-01-26

**Authors:** Qian Ke, Yongze Xu

**Affiliations:** 1 Faculty of Psychology, Beijing Normal University, Beijing 100875, China; 2 Department of Psychology, Faculty of Arts and Sciences, Beijing Normal University, Zhuhai 519087, China; 3 Beijing Key Laboratory of Applied Experimental Psychology, National Demonstration Center for Experimental Psychology Education (Beijing Normal University), Faculty of Psychology, Beijing Normal University, Beijing 100875, China

**Keywords:** employee turnover, turnover prediction model, text feature, professional networking platform, survival analysis

## Abstract

This study presents a novel methodology that integrates Transformer-based textual analysis from professional networking platforms with traditional demographic variables within a survival analysis framework to predict turnover. Using a dataset comprising 4087 work events from Maimai (a leading professional networking platform in China) spanning 2020 to 2022, our approach combines sentiment analysis and deep learning semantic representations to enhance predictive accuracy and interpretability for HR decision-making. Methodologically, we adopt a hybrid feature-extraction strategy combining theory-driven methods (sentiment analysis and TF-IDF) with a data-driven Transformer-based technique. Survival analysis is then applied to model time-dependent turnover risks, and we compare multiple models to identify the most predictive feature sets. Results demonstrate that integrating textual and demographic features improves prediction performance, specifically increasing the C-index by 3.38% and the cumulative/dynamic AUC by 3.43%. The Transformer-based method outperformed traditional approaches in capturing nuanced employee sentiments. Survival analysis further boosts model adaptability by incorporating temporal dynamics and also provides interpretable risk factors for turnover, supporting data-driven HR strategy formulation. This research advances turnover prediction methodology by combining text analysis with survival modeling, offering small and medium-sized enterprises a practical, data-informed approach to workforce planning. The findings contribute to broader labor market insights and can inform both organizational talent retention strategies and related policy-making.

## 1. Introduction

In the contemporary business environment, talent is widely recognized as an organization’s most valuable asset (Gupta & Sharma, 2022). Despite advancements in predictive analytics for turnover, significant challenges persist. For instance, the outcomes of predictive analyses often lack clear explanations, complicating informed decision-making for managers (Yuan et al., 2024). Furthermore, many models are static and cannot adapt to dynamic workplace conditions, compromising their prediction accuracy. Therefore, developing a robust and dynamic turnover prediction model remains a crucial challenge in human resource management.

This study synthesizes key established methods to develop a comprehensive model for predicting turnover. By mining user profiles and textual data from professional networking platforms, we obtained a large-scale and diverse sample capturing users’ career trajectories and attitudinal shifts. This approach thus offers more comprehensive and timely empirical support for predicting turnover. Additionally, this study validates relevant theories and advances understanding of the psychological drivers underlying turnover behavior. In developing the turnover prediction model, we prioritize both predictive accuracy and the identification of specific factors contributing to turnover risk.

This study has practical implications for organizational management. Specifically, by optimizing the prediction models, enterprises can enhance their HR systems (Yuan et al., 2024). This is particularly beneficial for small and medium-sized enterprises (SMEs), as it helps them overcome HR data constraints and leverage big data for accurate turnover prediction. By forecasting turnover risk within specific timeframes, such models provide enterprises with actionable insights to inform talent retention strategies (Min et al., 2024).

Beyond demographic features, this study incorporates textual information from professional networking platforms. This offers companies a new perspective on turnover, helping them understand employees’ genuine perspectives and core needs. Notably, the practical implications of this study extend from the organizational to societal and individual levels. The widespread application of such models facilitates a comprehensive analysis of turnover behavior across society. This enables government and related departments to stay informed about labor market developments, formulate accurate talent policies, and provide policy support to promote social stability and sustainable economic development (Juvitayapun, 2021).

The objective of this study is to develop an effective turnover prediction model by mining employees’ publicly shared textual data from professional networking platforms. The study comprises three core tasks: data acquisition, feature extraction, and modeling.

### 1.1. Data Acquisition

The Maimai platform, a widely used professional networking platform in China, was selected as the primary data source due to its large user base, frequent updates, and diverse features.

### 1.2. Overview of the Predictive Framework

Initially, demographic features identified as relevant in previous studies were selected to construct a baseline turnover prediction model. To enhance prediction accuracy, post data were also mined via two approaches, and their effectiveness was compared:

Theory-Based Approach: This approach extracts features using sentiment analysis and the TF-IDF method, based on a workplace lexicon.

Data-Driven Approach: This approach employs a Transformer-based semantic representation framework to extract text vectors.

### 1.3. Modeling

Survival analysis was selected as the modeling method because it utilizes temporal information (i.e., the influence of time on turnover probability) and handles censored data—a common occurrence in real-world turnover research. This method serves as the core analytical framework; various features are incorporated to construct multiple distinct models. Comparing these models provides insights into the most effective features for predicting turnover risk.

In summary, this study focuses on extracting demographic and textual features from professional social platforms and integrating them with survival analysis to construct a robust turnover prediction model. The remainder of this paper is organized as follows: Section 2 reviews advanced research in the field; Section 3 details the predictive analysis methodology; Section 4 and Section 5 present the key findings and how the company could use these insights to inform managerial decisions; and finally, Section 6 concludes the study.

## 2. Related Works

### 2.1. Turnover Theory

Traditional turnover theory posits a robust correlation between job-related, organizational, and individual psychological factors and turnover behavior. Numerous studies have identified key indicators of turnover risk, including job satisfaction, compensation, promotion opportunities, interpersonal relationships, work environment, and work–life balance (Saradhi & Palshikar, 2011; Gupta & Sharma, 2022; Mohiuddin et al., 2023). Ghosh et al. (2013), analyzing turnover interview data, found that a sense of professional responsibility or moral obligation drove employees’ affective attachment to the organization and intention to stay. Lloyd et al. (2015) highlighted the mediating role of positive affect in the relationship between the high-commitment HR system and turnover intention. Further, perceptions of fairness, workload, overtime, and job stress were significantly correlated with turnover propensity (Yuan et al., 2024).

The traditional approach to turnover modeling, which integrates qualitative and quantitative surveys—including interviews and supplementary investigations—retains significant limitations. First, the result of multivariate analyses heavily depends on data quantity and quality. In practical applications, issues such as incomplete data collection, sampling bias, and errors can compromise the accuracy of findings (Cai et al., 2020). Second, empirical research substantiating the predictive validity of these features is limited. Third, the absence of a unified theoretical framework hinders the development of a turnover model, leading to significant discrepancies in variable selection and methodologies. Finally, this lack of consensus complicates the formation of a comprehensive understanding, as various studies may yield divergent conclusions (Cohen et al., 2016).

This study conceptualizes language features from professional platforms as dynamic behavioral indicators of work attitudes and turnover propensity. The predictive validity of these features is grounded in established organizational theories. The Theory of Planned Behavior posits that attitude, subjective norms, and perceived behavioral control shape behavioral intention. Employees’ public expressions on platforms may reflect their attitudes towards work and perceived professional norms (Ouyang et al., 2023). The Social Exchange Theory underscores reciprocal relationships between employees and organizations. Expressions of negative affect or career exploration on platforms may signal perceived imbalances in this reciprocal exchange (Van Knippenberg et al., 2007). The Impression Management Theory highlights that even on professional platforms, self-presentation is strategic. Thus, language choices may reflect genuine turnover intentions or career self-promotion. Consequently, platform language is not mere ‘noise’ but meaningful digital behavioral traces embedded within complex socio-psychological processes, offering predictive value for turnover research (Van Dijck, 2013).

### 2.2. Construction of Turnover Prediction Model

Unlike traditional social media, professional networking platforms focus on career-related topics. Users on these platforms are authenticated using their real names. Compared to intra-enterprise HR data, data from these platforms are instantaneous and dynamic, reflecting real-time developments and opinions (Sharada et al., 2012), thereby providing timely empirical support for turnover research.

Additionally, demographic features are commonly used in turnover behavior research due to their strong predictive validity (Cho & Lewis, 2012). Factors such as age, gender, education level, years of experience, historical job changes, industry, and position shape work attitudes and decisions to leave. Sajjadiani et al. (2019) found that gender significantly predicts turnover, with female turnover rates higher than male turnover rates. Industry differences also affect turnover rates, with the internet industry showing higher turnover, especially in technical positions. The combined effect of education level and work experience is noteworthy: employees holding master’s degrees or higher show stronger turnover intentions after three to five years (Jain, 2017; Zhu et al., 2019; Priambodo et al., 2022). De Jesus et al. (2018) incorporated work experience hours as a predictive feature in turnover models, achieving 88% accuracy. These features were extracted from the original dataset and then coded or directly input to create a model-ready format.

Text mining techniques have facilitated a growing body of research on extracting valuable textual features from employees’ written expressions. Analyzing textual data, such as work emails and chat logs, yields insights into communication patterns, sentiment tendencies, and other work-related dynamics (Korytkowski et al., 2023). For instance, Sharada et al. (2012) developed a framework for predicting personality traits from social networking texts, while Qiao et al. (2019) created a Chinese corpus for competency analysis and applied deep learning models to text classification. Goldberg and Zaman (2018) analyzed employees’ sentiment tendencies toward human resource management by building a sentiment lexicon from social media comments. These studies demonstrate that analyzing textual data allows researchers to capture employees’ psychological states, behavioral patterns, and organizational dynamics more accurately. Specifically, these features are extracted through various machine learning and deep learning methods. Techniques such as PCA, self-encoders, and CNNs capture complex patterns and associations, extracting features with higher predictive power. For example, the word vector approach has been used to extract semantic representations from HR text data to predict employees’ Big Five personality scores (Guo et al., 2024).

Notably, focusing on the specific timing of turnover holds greater practical significance than the binary prediction of whether an employee will leave. Employee turnover is typically not an impulsive decision but a process that evolves (Kammeyer-Mueller et al., 2005). This complexity necessitates models that can handle continuous time features and accurately predict the exact timing of turnover events.

### 2.3. Survival Analysis in Turnover Prediction

Survival analysis models can incorporate multiple risk factors influencing turnover timing, thereby generating more nuanced predictions. For instance, factors such as age, job performance, and work environment are analyzed to identify employees more likely to leave within a specific future period. This enables targeted interventions. Additionally, survival analysis can handle data from employees who have not yet left by the study’s end but may do so in the future, a limitation that traditional classification models struggle with and which survival analysis mitigates through rigorous methodology.

For example, in a 13-year longitudinal study, Mulla et al. (2023) used survival analysis to identify factors influencing engineer turnover in a large public sector company in India. Li et al. (2017) proposed a novel survival analysis method for turnover prediction based on Cox proportional hazards (CoxPH) models, incorporating multitask learning to enhance predictive accuracy significantly. Jin et al. (2020) employed a random survival forest (RSF) model for turnover prediction, outperforming traditional machine learning models. Collectively, these studies illustrate the efficacy of survival analysis for turnover prediction, highlighting its ability to deliver actionable insights and improve organizational decision-making for employee retention strategies.

### 2.4. Survival Analysis Prediction Models

Survival analysis prediction models can be broadly classified into two categories: traditional survival analysis models and modern data-driven models.

Traditional survival analysis models are based on statistical principles, modeling the distribution of survival time data to estimate key metrics such as the survival and hazard functions. The Cox proportional hazards (CoxPH) model is the most representative and widely used in this category. As a semi-parametric model, the CoxPH does not assume a specific distribution for survival time but estimates the hazard function in a proportional form. Its core assumption is the proportionality of hazards across individuals, endowing the model with flexibility and robustness. It accommodates various covariates, data structures, and complex scenarios—including time-dependent covariates and time-varying effects—without requiring specific assumptions about the distribution of survival time.

However, the CoxPH model has limitations. The proportional hazards assumption may not hold in practice, especially when time-dependent effects are present. Moreover, issues such as collinearity and difficulties with model selection can arise with multiple covariates. Notably, its capacity to process high-dimensional data and capture complex nonlinear relationships is limited.

Modern Data-Driven Models have emerged with advances in machine learning and deep learning. These models excel at handling complex data structures and nonlinear relationships, improving prediction accuracy and flexibility. One representative method is the RSF.

RSF advantages include handling complex nonlinear relationships and high-dimensional data, as well as requiring fewer assumptions about data distribution. Additionally, RSF provides variable importance scores, aiding in identifying key factors influencing event timing. When relationships between predictors and event status are nonlinear and complex, RSF has demonstrated superior predictive performance compared to traditional regression methods (Ishwaran et al., 2021). However, as with other machine learning algorithms, RSF performance is dependent on data quality and parameter settings, requiring careful tuning and optimization in practice.

### 2.5. Evaluation Metrics

Survival analysis offers a range of evaluation metrics. Prediction accuracy is paramount, as it directly impacts the model’s practical application. Accuracy determines a model’s utility and reliability, making it the most crucial evaluation criterion. This study employs three widely recognized metrics in survival analysis: the concordance index (C-index), the integrated Brier score (IBS), and the area under the time-dependent ROC curve (AUC).

**C-index.** The C-index quantifies the consistency between the model’s predictions and observed event times (i.e., the accuracy of the model’s ranking of individuals by risk). It ranges from 0 to 1, with higher values indicating better performance. A C-index of 0.5 or lower suggests poor predictive discrimination.

**IBS.** IBS extends the Brier score to accommodate right-censored data, which is particularly useful for assessing calibration. It measures mean-squared differences between predicted turnover probabilities and actual turnover events at specified time points (Ishwaran et al., 2021). Values range from 0 to 1, with lower values indicating better model calibration. An IBS value of 0.25 or higher suggests inadequate predictive capability. Applying IBS yields a comprehensive understanding of model performance across time points, providing crucial insights for further optimization.

**Time-dependent AUC (mean_AUC).** Due to the time-dependent nature of survival data, traditional ROC curves are not directly applicable. Instead, cumulative/dynamic AUC metrics are used (Hao et al., 2021). The mean_AUC value over a specified period ranges from 0 to 1, with higher values indicating stronger overall discriminative ability.

These metrics collectively enable a thorough assessment of the predictive performance and robustness of survival analysis models, guiding model improvements and ensuring practical applicability.

## 3. Materials and Methods

### 3.1. Dataset

The raw data for this study were sourced from Maimai, a widely used professional networking platform in China known for its vast user base. The platform offers comprehensive demographic information, including work experience, occupational labels, and educational background, making it an ideal source for turnover prediction studies (Zheng et al., 2022). Personal public data from real-name-authenticated users was accessed using Python 3.9.12, in compliance with legal regulations and the platform’s terms of service, thereby ensuring the legality and validity of the data. Each user record consists of two main components: basic demographic information and post content. The former encompasses six dimensions, including nickname, gender, city, total number of postings, education experience, and work experience, while the latter contains all text posted by users, offering insights into their sentiment, job satisfaction, and organizational attitudes during work events.

To protect user privacy, all data were anonymized and stored in CSV format, facilitating analysis while complying with data privacy and security regulations.

Employees may exhibit varying behaviors and decisions over time and across organizational contexts (Zhu et al., 2019).

This study specifically investigates the predictive value of linguistic traces from professional social platforms. Consequently, our analysis is intentionally focused on a sample of users who have generated at least one post during their work event. Work events with zero user-generated posts were therefore excluded at the data collection stage.

We acknowledge that this defines a cohort of “active posters”. This focus is methodologically necessary to address our core research question regarding textual data. To empirically characterize this “active poster” cohort, we provide descriptive statistics for their platform activity. The distribution of posting frequency per work event was right-skewed, with a median of 7 posts (interquartile range [IQR]: 2–25). This indicates that our analytical sample is centered around users with sustained platform engagement while also encompassing a wide range of activity levels. However, it limits the generalizability of our findings to the broader employee population, including low- or non-active platform users, should the determinants of turnover differ between these groups. The implications of this sample selection for interpretation are further discussed in the Limitations section (Section 6.1). While our sample is restricted to active posters, the demographic profile (e.g., industry distribution, regional composition) of our cohort is broadly aligned with the platform’s reported overall user demographics (Maimai & Yiguan, 2018). This suggests that the selection bias may be more pronounced along the dimension of online engagement behavior than along these basic structural demographic lines.

#### 3.1.1. Definition of Work Events and Data Processing

In this study, each “work event” was treated as a distinct analytical unit. A work event was defined as a continuous period of employment at a single company, as documented in a user’s profile. For each work event, the prior work event duration was calculated as the time from the start of the user’s earliest job to the start of the current work event. Similarly, the total number of prior work experiences was counted to the beginning of that event. The number of user-generated posts during the current work event was aggregated, excluding instances with zero posts.

#### 3.1.2. Inferring Turnover Events from Work History

Turnover events were inferred from the user’s self-reported work history. Each work history entry includes a start date and, where applicable, an end date. Turnover was operationalized as a work event with a recorded end date falling within our observation window (1 January 2020 to 31 December 2022). This end date was treated as the turnover date. We acknowledge that this method is susceptible to measurement error due to potential delays or infrequent user profile updates, meaning the actual departure may have occurred earlier than the date recorded. Consequently, work events without a recorded end date were treated as ongoing and coded as censored observations.

#### 3.1.3. Data Processing and Cleaning Procedure

The following steps were taken to process and clean the data:

**Extraction:** Start (*start_date*) and end (*end_date*) dates were extracted from the “work experience” section of each user profile. As the original data provided granularity only to the year and month (e.g., “2021-08”), all dates were standardized to the *YYYY-MM* format and treated as representing the first day of the month (e.g., “2021-08-01”) for computational consistency.

**Handling Missing End Dates:** Work entries with a missing *end_date* were considered to represent current employment at the time of data collection. For these entries, the *end_date* was set to NULL.

**Alignment with Observation Window:** To define a consistent analysis cohort, the observation period for each work event was determined as:

1. Observation Start Date (*obs_start*): If the work event’s original *start_date* was before 1 January 2020, obs_start was set to 2020-01-01. Otherwise, *obs_start* was set equal to the original start_date.
2. Observation End Date (*obs_end*) and Event Status:
  - If a work event had a recorded *end_date* within 2020-01-01 and 2022-12-31, then *obs_end* was set to the recorded *end_date*, and the event status was coded as 1 (*turnover*).
  - If a work event had no recorded *end_date* or its recorded *end_date* was after 2022-12-31, *obs_end* was set to 2022-12-31, and the event status was coded as 0 (*censored*, indicating no observed turnover within the window).

**Figure 1:**
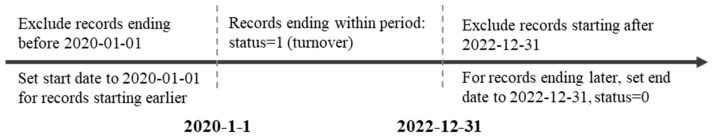
Data Processing and Event Status Coding Workflow.

Work events with a recorded *end_date* before 2020-01-01 were excluded because they ended before our observation window. Figure 1 presents the workflow for data cleaning and processing. Table 1 illustrates the process of mapping a single user profile to multiple work events and the subsequent coding of turnover/censoring status based on our rules.

#### 3.1.4. Calculation of Retention Time

The retention time for survival analysis was computed as the difference in months between the observation end time (*obs_end*) and the observation start time *(obs_start*). This variable represents the duration (in months) a work event was under observation until either turnover occurred or the study period ended.

#### 3.1.5. Dataset Preparation

During dataset preparation, missing values were addressed using methods such as mode imputation, and formatting errors were corrected. The final analytic dataset comprised 4087 work events, including 1153 turnover events, yielding a censoring rate of 71.79%.

Text data from the corresponding 4078 work events (excluding 9 cases with unprocessable text) underwent cleaning, segmentation, and stop-word removal. This involved eliminating non-Chinese characters, special symbols, and irrelevant characters, and standardizing formatting by removing redundant spaces and line breaks. Texts were segmented using the Jieba tool with a custom lexicon for proper nouns. A comprehensive stop-word list was compiled by integrating lists from authoritative sources (e.g., HIT, Sichuan University, Baidu) and applied to the corpus, yielding 208,204 textual entries corresponding to the work events.

### 3.2. Ethical Considerations

This study utilized publicly available profile data from the Maimai platform in full compliance with its Terms of Service and data access protocols. Throughout the data collection, no private messages or non-public information were accessed. To protect user privacy, all personally identifiable information (PII), such as nicknames, specific company names, and detailed city locations, was removed or generalized during data processing. Company names were replaced with industry categories, and cities were aggregated into broader regions. Although the raw text of user posts was retained for feature extraction, it was permanently decoupled from direct user identifiers before analysis. The final dataset used in this study consists only of derived, anonymized, and aggregated features.

Given the public nature of the source data and the aggregation measures implemented, the risk of re-identifying individuals from the processed dataset is considered minimal. This research analyzed publicly accessible professional profile data, for which users have a limited expectation of privacy. As the study involved no interaction with human subjects and focused on aggregate patterns, Formal Institutional Review Board (IRB) approval and individual informed consent were not required, in accordance with established ethical guidelines for research involving publicly available data (Egelman et al., 2012; Van Dijck, 2013).

### 3.3. Feature Extraction

#### 3.3.1. Demographic

In this study, eight independent variables (predictors) were selected: gender, number of posts, years of work experience, number of previous jobs, city, highest education level, industry, and position. Retention time (in months) and event status were selected as outcome variables for model construction. The “number of postings” was included based on the hypothesis that highly active users during their employment tenure might be more inclined to reveal turnover-related information. The predictive validity of the remaining seven variables has been established in prior research.

Specifically, for city location, we referenced the 2021 economic belt classification by China’s National Bureau of Statistics (NBS) to categorize each city into its respective regional grouping. The top five industries and occupations with the highest sample share were selected for detailed analysis.

Subsequently, One-Hot Encoding was employed to process categorical data, and numerical data were normalized (a linear transformation technique). Table 2 summarizes the final set of 24 features.

#### 3.3.2. Textual

Two approaches, theory-oriented and data-oriented, were employed to extract text features. The theory-oriented approach relies on existing psychological theories, employing sentiment analysis and lexicon-based TF-IDF for topic extraction to interpret textual content. Sentiment analysis captures emotional tone and intensity, elucidating individuals’ emotional attitudes and psychological states. In contrast, data-oriented methods rely on Large Language Models (LLMs) to learn and represent semantic information without predefined theoretical frameworks. Unlike the lexicon-based TF-IDF method for topic extraction, this approach enables data-driven identification of key topics, uncovering latent semantic patterns and relationships.

The theory-oriented framework offers greater interpretability, while the data-oriented framework emphasizes predictive performance. Additionally, given the potential impact of temporal changes on turnover behavior, textual features were aggregated within each work-event period using cumulative weights. The weight of each text was determined by its proximity to the end time of the work event: the closer the proximity, the higher the weight. This method mitigates the interference of temporal factors on turnover prediction results. The key processing steps are outlined below:

Let *i* represent the reverse index position of a text within the same work event, indicating the *w_i_*, which is inversely proportional to the distance from the end time of the work event. This weight decreases gradually as the distance from the end time of the work event increases. This weight serves as the individual feature value for the respective text.

(1) $$w_{i}=\frac{1}{i+1}$$

The weighted feature value can be expressed as:

(2) $$f_{i}^{\prime }=w_{i}\times f_{i}$$

For all texts within the same work event period, the merged individual feature is:

(3) $$F_{merge}=\Sigma_{i}f_{i}^{\prime }$$

**Sentiment scores.** Sentiment scores were extracted using the senta_lstm pre-trained model accessed via the PaddleHub toolkit within the PaddlePaddle framework. This model employs an LSTM network architecture effective for capturing contextual dependencies in sequence data, making it suitable for sentence-level sentiment analysis of Chinese text. We loaded the model directly using the *hub. Module (name = “senta_lstm”)* and applied its sentiment classification function to each user’s post to obtain probabilistic sentiment predictions. For each text, the model outputs a confidence score for both positive and negative sentiment polarities (Fang et al., 2023). Two aggregated features were subsequently derived as inputs for the predictive model: the positive sentiment score (*Positive_pro*) and the negative sentiment score (*Negative_pro*) for each work event, calculated by averaging the corresponding probabilities of all posts within that event period (subject to the time-based weighting scheme described in Section 3.3.2).

**Topic scores.** We employed the TAPS lexicon developed by Speer et al. (2023). This lexicon is a text-based attitude and perception scale derived from detailed questionnaires and interviews with 1506 employees of small- and medium-sized enterprises regarding their work attitudes and perceptions. This systematic curation process yielded a workplace lexicon containing 22 themes related to work attitudes and perceptions. To adapt the lexicon to the Chinese context, the original lexicon underwent a rigorous localization process: it was first translated into Chinese independently by two bilingual researchers, with discrepancies reconciled by a third. This translated version was then expanded using the HIT thesaurus (Fu et al., 2013), which was used for effective lexical expansion, while irrelevant or repetitive words, as well as content involving racial discrimination, religion, etc., were removed to better fit the Chinese workplace environment. The final workplace lexicon comprised 22 categories and 7786 words. The TF-IDF value of each text in the dataset was calculated for each category in the lexicon and all corresponding words. The overall TF-IDF score for each text in each topic category was derived and termed the “topic score.”

**Text vector.** Text was first input into the pre-trained ERNIE 3.0-medium model (Guo et al., 2024). ERNIE 3.0 has demonstrated enhanced performance on Chinese NLP tasks, including sentiment analysis, reading comprehension, and named entity recognition (Zheng et al., 2022). Compared to the BERT Base model, ERNIE 3.0, with twice the parameter size, achieves a 2.5% improvement in accuracy and a 3.4% improvement over the RoBERTa Base of the same size. We used the model in its default configuration with a maximum sequence length of 512 tokens and extracted the pooled output of the final layer as the sentence representation. This yielded a 768-dimensional vector capturing the overall semantic information of the entire text. To mitigate potential risks such as model overfitting caused by high-dimensional features, PCA was employed as a dimensionality reduction technique. Critically, the PCA transformation was fitted exclusively to the training set. This allowed the projection of the original high-dimensional data into a low-dimensional space while preserving the principal features and ensuring no information leakage from the test set. The feature set was ultimately reduced to 11 dimensions by retaining components that cumulatively explained 60% of the variance.

### 3.4. Model Construction

In this study, CoxPH and RSF models were used for analysis. The dataset was randomly split into a training set (80%) and a held-out test set (20%). All data preprocessing steps, including normalization of demographic features and fitting the PCA transformation for text vectors, were performed using the training data only. The fitted transformers were then applied to the test set to prevent data leakage. Model hyperparameter tuning for RSF was conducted strictly within the training set using 10-fold cross-validation. The final model was evaluated only once on the held-out test set. Statistical significance of performance differences between models or feature sets was assessed using paired *t*-tests on the concordance indices of the 10-fold cross-validation replicates. To report stable metrics, this entire train–test split procedure was repeated 10 times with different random seeds, and the mean performance metrics were calculated.

The preprocessed demographic and textual features described earlier were incorporated to construct prediction models with different feature combinations. These models were then comparatively evaluated and analyzed. Table 3 provides details of the prediction models corresponding to each specific feature combination.

Furthermore, for consistency and comparability, all models used identical training and test set divisions based on work events. Additionally, both modeling frameworks, CoxPH and RSF, were used uniformly across different models, with the only variation in the input feature sets.

## 4. Results

Diagnostic checks were conducted on the CoxPH models. The proportional hazards assumption was assessed using Schoenfeld residuals (via the *lifelines* package), with no significant violations detected at a global significance level of 0.05. Tied event times were handled using the Efron approximation, the default method in the employed statistical package.

Performance metrics were calculated for both the CoxPH and RSF models across different feature combinations. Improvements in the C-index, IBS, and mean_AUC relative to the Base model (relying solely on demographic features) were also compared. Higher values of the C-index and mean_AUC indicate better predictive performance, while lower IBS values indicate superior performance. Results are summarized in Table 4 and Table 5. Given the high stability of the IBS values across all prediction tasks and models (overall variation < 0.05%), predictive accuracy was primarily evaluated and compared using the C-index and mean_AUC. Subsequent analyses focus on three aspects: model selection, the effectiveness of incorporating textual features, and the choice of text feature extraction approaches.

Model selection was guided by both performance metrics and information criteria. The semi-parametric CoxPH model allows for evaluation of parsimony using AIC and BIC, whereas the non-parametric RSF was primarily evaluated based on its cross-validated predictive performance (i.e., C-index, IBS, mean_AUC).

### 4.1. Textual Feature Integration Effect

Comparison across models reveals that the combination of text vectors and sentiment scores (Model 5) constituted the optimal feature set for predicting turnover.

In the CoxPH framework, Model 5 achieved a C-index of 0.7313 and a mean_AUC of 0.7899. These represent improvements of 3.33% and 3.09%, respectively, over the demographic-only baseline (Model 1). Similarly, within the RSF framework, Model 5 attained the highest C-index (0.7514) and mean_AUC (0.8071), corresponding to improvements of 2.82% and 2.68%, respectively.

Although the model incorporating all textual features (Model 7) showed nearly identical predictive performance, Model 5 was selected as the optimal due to its greater parsimony. This is evidenced by its lower AIC and BIC values in the CoxPH, indicating a more efficient balance between model complexity and goodness-of-fit while maintaining high accuracy.

### 4.2. Comparison of Textual Feature Extraction Methods

Text vector contributed most significantly to the prediction models. Adding the text vector resulted in improvements exceeding 2.8% for both the CoxPH and RSF models compared to the baseline. Introducing sentiment scores also improved the C-index and mean_AUC values relative to the baseline.

However, introducing topic scores alone produced no significant improvement in the C-index values for either model (*p* > 0.05, paired *t*-test). Although combining this feature with others slightly enhanced performance, the improvement was not statistically significant compared to models excluding the topic scores (*p* > 0.05). Therefore, this study does not recommend using lexicon-based theme analysis for turnover prediction in this context.

In conclusion, models incorporating text vectors and sentiment scores demonstrated better performance in predicting employee turnover. Text feature extraction methods, particularly those relying on lexicon-based theme analysis, may require further refinement to improve their effectiveness in diverse workplace contexts.

### 4.3. Model Optimization

Model 5 achieved the highest prediction performance, making it the optimal choice for further refinement. Its superiority was confirmed by the feature importance ranking derived from Mean Decrease Impurity (MDI) analysis in the RSF framework. 23 features were retained, all of which improved prediction accuracy (MDI > 0).

To optimize the RSF model, the following hyperparameters were fine-tuned:

***n_estimators***: Number of trees in the forest.

***max_depth***: Maximum depth of the trees.

***min_samples_leaf***: Minimum number of samples required to be at a leaf node.

**Figure 2:**
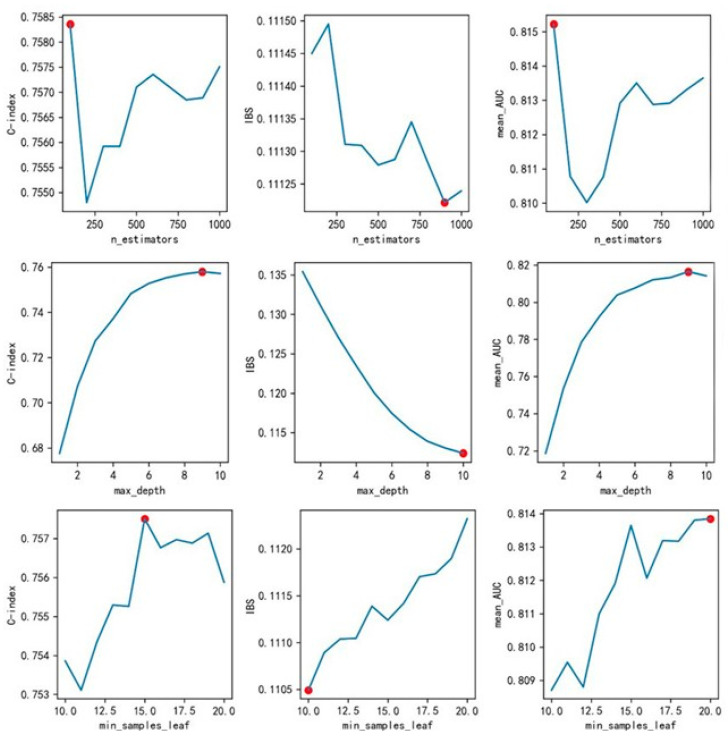
Trend of predictive indicators under each parameter of the RSF model. The red dots indicate the optimal values selected for the final model.

A sequential tuning strategy was employed, testing each parameter within the following ranges using grid search and 10-fold cross-validation: *n_estimators* [100, 1000], *max_depth* [1, 10], *min_samples_leaf* [10, 20]. The predictive performance of models with the selected features across different parameter values is visualized in Figure 2.

The C-index, IBS, and mean_AUC did not consistently achieve optimal values across all parameters. Given the C-index’s broad acceptance as a metric reflecting the ranking accuracy of risk predictions, we prioritized its improvement during optimization, while also considering trends in other metrics.

For *n_estimators*, the optimal C-index and mean_AUC values were observed at 100, but IBS was the worst at this value. Therefore, we selected *n_estimators* = 1000, which offered a slightly lower C-index and mean_AUC but significantly better IBS.

For *max_depth*, C-index, and mean_AUC, 9 was optimal. Although IBS was not optimal at this value, the difference was minimal; thus, we chose *max_depth* = 9.

For *min_samples_leaf*, optimal parameter values varied across indicators. We chose *min_samples_leaf* = 15, which yielded the best C-index performance and relatively strong results for other indicators.

Based on the comparison of model performance across parameter combinations, we selected the final configurations: *n_estimators* = 1000, *max_depth* = 9, *min_samples_leaf* = 15, and *random_state* = 20.

Following feature selection and parameter tuning, the RSF model showed further improvement on the test set. Results are presented in Table 6. In summary, optimizing Model 5 through careful parameter tuning of the RSF method resulted in significant improvements in prediction accuracy, demonstrating the value of integrating textual features and fine-tuning machine learning models for turnover prediction.

## 5. Discussion

### 5.1. Impact of Demographic Features on Turnover Behavior

**Figure 3:**
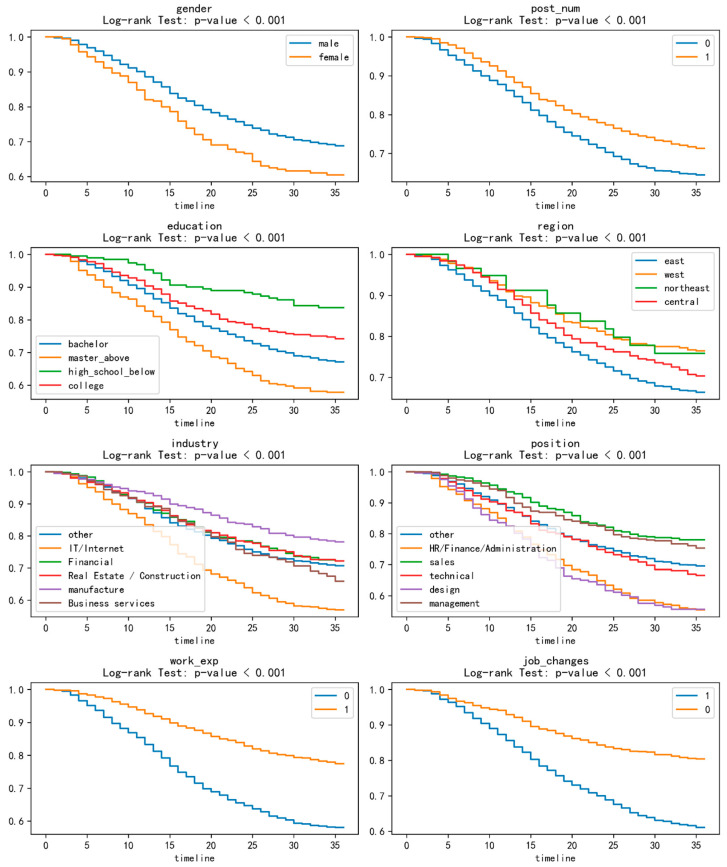
Survival curve for each categorical feature.

To examine the impact of demographic features on turnover behavior, the Kaplan–Meier (KM) curve method was employed to compare the survival curves across different categories. The log-rank test was used to assess significant differences in retention rates across subgroups. For numerical features, stratification was based on the median: values below the median were coded as 0, and values at or above the median were coded as 1. Survival curves for all demographics are shown in Figure 3.

**Gender.** Regarding gender, the retention rate of female employees was consistently lower than that of male employees at most time points. This aligns with Cho and Lewis (2012), who observed that women are less attached to the labor market and thus more likely to turnover than men. However, this finding may be subject to uncertainty due to the relatively small number of female employees in the study sample (the male-to-female ratio is close to 9:1), suggesting that this difference could stem from sample imbalance.

**Social Media Activity.** Employees who posted frequently on the professional platform exhibited a lower turnover risk. This may indicate higher levels of work commitment and job satisfaction. These employees utilized the platform to demonstrate their professional competence, work achievements, and career attitudes—factors that enhance their job stability and satisfaction. Their strong willingness to stay is reflected in their active posting, creating a virtuous cycle.

**Education.** Overall, employees with higher education levels had lower retention rates. The survival curve for the group with a high school education or below was relatively flat, suggesting that employees with lower educational attainment are more likely to gain job satisfaction and organizational loyalty, thereby maintaining stable retention over time. Conversely, highly educated employees tend to have higher job expectations; dissatisfaction with the current work environment may increase their turnover risk. This is consistent with Chang et al.’s (2008) finding that R&D employees with doctoral degrees had higher turnover rates than those with bachelor’s degrees.

**Region.** Turnover rates in the eastern coastal economic zone were significantly lower than in other regions. This finding is consistent with previous research indicating that turnover rates are usually lower in more economically developed regions (Zhu et al., 2019). Overall, rates in other regions did not differ significantly, potentially due to the relatively small number of employees in these regions (representing only 23.22% of the total sample).

**Industry.** The retention rate in the IT/Internet industry after one year was significantly lower than in other industries. In contrast, the downward trend in the manufacturing industry was relatively flat. This may be attributed to the high competitiveness, intense work pressure, and youthful work culture characteristic of the Internet industry. Conversely, the stability and maturity of the manufacturing industry, the availability of training opportunities, and the industry’s dependence on economic conditions and market demand may contribute to its lower turnover rates.

**Position.** A key finding was inconsistent with previous research: design and functional positions had significantly lower retention rates than other positions, while sales and senior management positions had relatively higher retention rates. This discrepancy may arise because the data captured only positions from users’ most recent work event, failing to reflect the status of their prior work event. Further analysis of the position status across an individual’s entire career sequence would be required to more accurately analyze the impact of positions on employee retention.

**Work Experience.** A negative correlation existed between employees’ years of work experience and their risk of turnover. Specifically, employees with more years of experience exhibited a lower turnover risk. This can be attributed to the strong sense of organizational belonging and loyalty that employees develop over time (Gupta & Sharma, 2022). Additionally, longer tenure may facilitate more favorable career development opportunities and benefits for employees, thereby reinforcing their willingness to remain with the organization.

**Job Changes.** Employees with a history of frequent job changes exhibited a higher risk of turnover. This may be directly related to career stability. Employees with frequent job changes are more likely to be tempted by external career opportunities or dissatisfied with their current work environment, leading to a higher turnover rate (Jayaratne & Jayatilleke, 2022).

### 5.2. Performance of Turnover Prediction over Time

**Figure 4:**
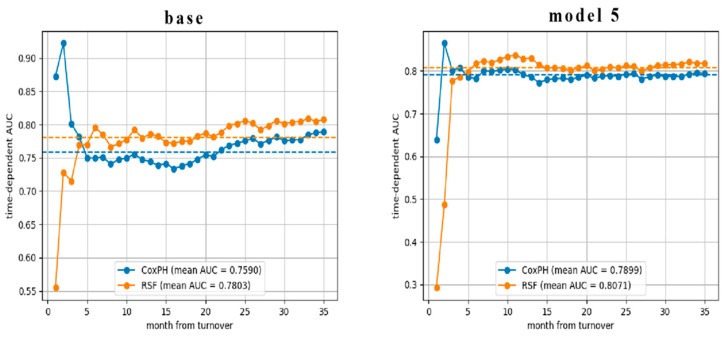
Trend curves of cumulative/dynamic AUC for the baseline model and Model 5. The dotted lines represent the mean AUC.

To investigate the model’s predictive performance over time, particularly during periods of rapid turnover, we compared the cumulative and dynamic AUC trend curves of the baseline model with those of the optimal model incorporating textual features (Figure 4).

#### 5.2.1. Initial Period (First Month)

Model 5 was less effective at distinguishing turnover cases among employees in their first month. This initial adaptation period involves complex factors influencing turnover that are not adequately captured in the platform texts. This complexity, coupled with limited textual information, results in lower predictive accuracy during this early phase.

#### 5.2.2. Early Employment Period (First Four Months)

During the first four months of onboarding, the CoxPH model outperformed the RSF model in terms of AUC. The baseline model performed similarly during this period. However, the model incorporating textual features performed relatively poorly in terms of AUC at this early stage.

Two main reasons may explain this:

CoxPH Model Robustness: The CoxPH model’s assumption of proportional hazard is more tenable in the early stages. Its use of partial likelihood estimation for censored data confers greater robustness with smaller datasets.

RSF Model Limitations: The RSF model may overfit due to limited data in the initial onboarding stage. The RSF method employs bootstrap sampling. In the early months, the high censoring proportion leads to sample imbalance, impairing the model’s ability to accurately reflect turnover risk. Additionally, bootstrap randomness can significantly affect prediction accuracy with small samples.

#### 5.2.3. Later Employment Period (From Fifth Month Onwards)

From month five onwards, the RSF model incorporating textual features consistently outperformed the CoxPH model.

The CoxPH model performed better at predicting turnover from 2 to 12 months after onboarding, with AUC values exceeding the overall average.

However, the RSF model showed favorable predictive performance from the fifth month onward. The model incorporating textual features maintained superior performance for a longer period than the baseline model. Notably, the overall AUC increased significantly from the sixth month onward, corresponding to the rapid turnover phase.

This result substantiates that incorporating textual features extends the effective prediction period for turnover.

### 5.3. Contribution of Various Features to the Predicted Results

**Figure 5:**
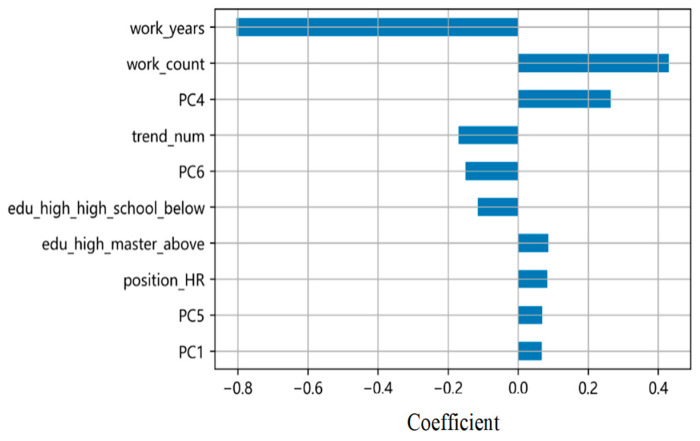
Top 10 CoxPH Best Predictive Model Coefficient Features.

**Figure 6:**
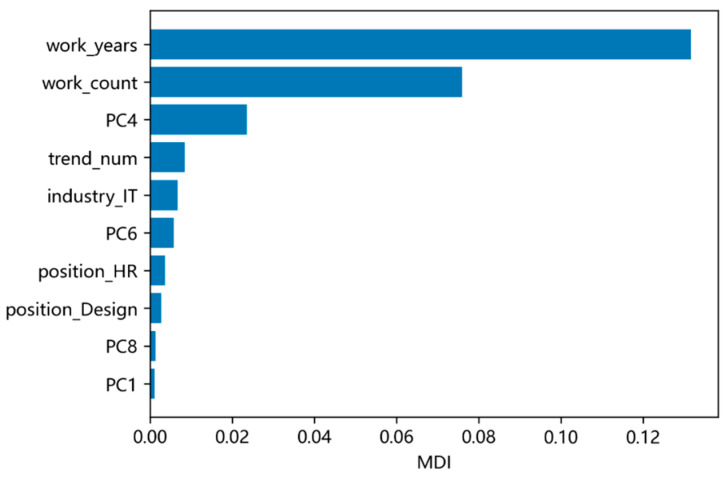
Top 10 RSF Best Predictive Model MDI Features.

To investigate the contributions, we ranked the top 10 risk regression coefficients for the optimal CoxPH model (Model 5; Figure 5) and the top 10 MDI features for the optimal RSF model (Model 5; Figure 6).

Comparative analysis indicated that demographic features accounted for approximately 60% of the top-ranked features in both models. This suggests that demographic features significantly impact turnover prediction. Notably, the following features were among the top-ranked in both models: years of work experience, number of previous work experiences, number of posts, and HR-related position.

Years of experience and the number of previous work experiences were particularly influential. Discrepancies in the ranked contributions of other demographic features may be attributed to differences in model sensitivity and interpretation.

Transformer-based semantic representation also played an important role, with PC4, PC6, and PC1 among the top-ranked features. Although these PCA-derived components are not directly interpretable as discrete topics, their high rankings indicate they capture systematic, predictive semantic patterns. These dense vectors likely function as efficient latent indicators of complex workplace attitudes (e.g., career focus, satisfaction, or stress) expressed diffusely in natural language but distilled by the model. Their value lies in providing a potent, data-driven complement to traditional structured predictors.

The absence of sentiment scores from the top individual feature rankings contrasts with the optimal performance of Model 5 (which integrated sentiment with text vectors). This suggests that the predictive power of sentiment did not manifest as a strong independent signal but emerged through synergistic interaction with other features, particularly the semantic context from text vectors. Therefore, the direct model comparison in Section 4.2 remains the primary evidence for identifying the optimal feature set (text vectors + sentiment). The feature importance analysis complements this by revealing that efficacy arises from feature interactions rather than the dominance of any single component.

## 6. Conclusions

This study employed survival analysis models to predict the turnover risk of employees over time, comparing demographic features with those incorporating textual features. The results established that the RSF model demonstrated superior fit and prediction accuracy on this dataset. Furthermore, incorporating textual features significantly enhanced overall model performance compared to using only demographic features. Among feature combinations, integrating text vectors and sentiment scores proved most effective.

### 6.1. Limitation

This study has several limitations, which also point to directions for future research. First, reliance on data from a single professional platform in China may limit the generalizability of findings to other cultural or industrial contexts. Relatedly, the significant gender imbalance and potential self-selection bias among active platform users suggest results may not fully represent the broader workforce.

Second, the textual data have inherent constraints. User-generated posts encompass a wide range of topics, including non-work-related content like news retweets and advertisements. This variety, combined with the curated nature of public personas, means the data may not perfectly align with private attitudes or capture all dimensions of turnover intention explored in traditional surveys. Future work would benefit from more refined topic filtering to isolate work-relevant signals.

Third, sample selection is inherently linked to platform activity. By design, our analysis focuses on “active posters” (users who posted at least once during a work event under observation; median = 7 posts, IQR: 2–25). We lack data on employees without platform activity. Consequently, our predictive model and the identified feature importance (e.g., posting frequency) are primarily applicable to this digitally engaged subset. More broadly, if the propensity to post correlates with unobserved traits (e.g., higher career mobility orientation), the model’s estimated relationships may reflect the dynamics within this subgroup. Therefore, the generalizability of findings, particularly the feature importance rankings, to entirely inactive employee populations is limited and remains an important question for future research. Studies with access to internal organizational data could validate whether the identified relationships hold for the broader workforce.

A related methodological limitation is the limited effectiveness of the theory-driven, lexicon-based topic extraction method. The pre-constructed dictionary, while theoretically grounded, may not have fully captured the evolving, context-specific language of the platform. This limitation could stem from several factors.

Localization issues: the dictionary, developed in a different cultural context, might not perfectly match the nuances of the Chinese professional online discourse.

The inherent difficulty of a static lexicon adapting to the dynamic language of large-scale user-generated content. This underscores the challenge of applying static dictionaries to dynamic, platform-specific language. It supports a shift toward more adaptive, data-driven semantic representations (e.g., Transformer models) or the development of dynamic, domain-specific lexicons in future research.

Finally, while we adhered to ethical data practices, the organizational application of such predictive models using public data necessitates ongoing consideration of privacy and consent. Future research should validate and extend this approach using multi-source, cross-cultural datasets; employ more advanced NLP techniques to interpret nuanced textual data; and engage with the ethical implications of leveraging digital traces for HR analytics.

### 6.2. Future Research Directions

Future research should explore different types of textual features and develop more effective extraction methods. This includes refining natural language processing techniques and topic classification methods to better integrate textual features with non-textual data (e.g., job performance metrics), thereby improving prediction accuracy.

Future studies should integrate multiple data sources to gain a more comprehensive understanding of employee behavior and psychological states. In addition to publicly available social media data, internal corporate communication and employee performance data should be analyzed. Examining the correlations among these sources and their value as early warnings of turnover risk could significantly enhance predictive capabilities.

The generalizability of models should be tested using data from various industries and organizations of different sizes. This will help assess model applicability in diverse practical settings. Additionally, exploring model transfer learning—where a model trained on data from one context is applied to others—could improve model robustness and utility.

Addressing these areas can lead to the development of more accurate and generalizable turnover prediction models, ultimately helping organizations better understand and mitigate employee turnover risks. The study’s primary contribution lies in its innovative methodological integration—combining advanced text mining with survival analysis—which addresses a significant gap in contemporary turnover prediction research.

### 6.3. Managerial Implications and Ethical Guidelines

This research bridges HR analytics and computational linguistics, demonstrating the predictive value of publicly available digital traces for understanding turnover dynamics. The findings offer a data-informed perspective for talent retention, yet their application must be guided by robust ethical frameworks to ensure responsible practice.

For practitioners, the model serves as a proactive tool for talent retention. Organizations can integrate such predictive insights into HR systems to identify employee segments at elevated turnover risk. This enables targeted, supportive interventions such as confidential stay interviews, career development conversations, or tailored support programs.

Managers should interpret these insights as indicators prompting further inquiry, rather than as definitive verdicts. Predictions must be complemented by direct communication and fair HR practices. Model outputs should be treated as early-warning indicators prompting further inquiry and support, not as assessments of employee loyalty. HR practitioners must interpret predictions within the broader individual and organizational contexts.

Ethical implementation requires adherence to key guidelines: The application of such models must prioritize ethical use.

Aggregated Application: Prioritize applying insights at a group level (e.g., to identify trends in departments or job families) to inform systemic improvements.

Transparency and Safeguards for Individual Use: If used to inform individual support, ensure transparency and implement clear safeguards.

Prohibition of Automated Decisions: the model must not be used for automated monitoring, profiling, or as the sole basis for automated personnel decisions.

Purpose Limitation: use analytics strictly to improve retention and organizational support, preferring aggregated, de-identified data where possible.

Human-in-the-Loop: Predictions require review by HR professionals to mitigate bias, ensuring final decisions involve human judgment.

Beneficial Purpose: The primary goal must be to provide better support or improve work conditions.

Supportive Interventions: Interventions should be supportive, not punitive, fostering trust and development.

**Theoretical Contribution.** For theory, this study extends turnover models by incorporating real-time, behavioral text data as a proxy for evolving job attitudes. By treating platform language as digital behavioral traces, it provides a new lens for examining employee–organization relationships in the digital age, thereby expanding the explanatory boundaries of social exchange and turnover process theories.

## Figures and Tables

**Table 1:** Illustration of data processing for a hypothetical user profile.

User ID	Original Start	Original End	Derived Work Event ID	Observation Start (*obs_start*)	Observation End (*obs_end)*	Retention Months (approx.)	Event Status	Notes
U123	2018-6-1	2020-8-15	WA1	2020-1-1	2020-8-15	7.5	1	Start truncated. Ended in the window.
U123	2020-9-1	2022-4-30	WB1	2020-9-1	2022-4-30	19.9	1	Fully within the window.
U123	2022/6/1	NULL	WC1	2022/6/1	2022/12/31	6.9	0	Ongoing job, right-censored

**Table 2:** Demographic feature description.

Name	Description
gender	Gender of employee
trend_num	Number of postings
work_years	Duration (years) of work experience prior to the start of the current work event
work_count	Count of work events occurring within the observation period
city_type_east_north	City Region—Northeast
city_type_east	City Region—East
city_type_mid	City Region—Central
city_type_west	City Region—West
edu_high_master_above	Highest level of education—Master’s degree and above
edu_high_bachelor	Highest level of education—Bachelor’s
edu_high_college	Highest level of education—Junior college
edu_high_high_school_below	Highest level of education—High school and below
industry_IT	Industry—IT/Internet
industry_Manufacture	Industry—Manufacture
industry_Business_services	Industry—Business services
industry_Real_estate	Industry—Real Estate/Construction
industry_Financial	Industry—Financial
industry_Other	Industry—Other
position_HR	Position—HR/Finance/Administration
position_Technical	Position—Technical
position_Design	Position—Design
position_Sales	Position—Sales
position_Management	Position—Management
position_Other	Position—Other

**Table 3:** Description of different models.

Model	Feature Number	Features Description
Baseline	24	Demographic
Model 1	26	Demographic/sentiment score
Model 2	46	Demographic/topic score
Model 3	35	Demographic/text vector
Model 4	48	Demographic/sentiment score/topic score
Model 5	37	Demographic/sentiment score/text vector
Model 6	57	Demographic/topic score/text vector
Model 7	59	Demographic/sentiment score/topic score/text vector

**Table 4:** Results of CoxPH turnover prediction models with different models.

Model	C-Index	Up	IBS	Up	mean_AUC	Up	AIC	BIC
Baseline	0.6981	/	0.1193	/	0.7590	/	49.406	195.620
Model 1	0.7024	0.0043	0.1189	0.0004	0.7624	0.0034	53.411	211.809
Model 2	0.6991	0.0010	0.1191	0.0002	0.7605	0.0015	93.407	373.650
Model 3	0.7309	0.0328	0.1149	0.0044	0.7897	0.0307	69.437	276.573
Model 4	0.7034	0.0053	0.1188	0.0005	0.7637	0.0047	97.413	389.840
Model 5	0.7313	0.0332	0.1149	0.0044	0.7899	0.0309	73.438	292.758
Model 6	0.7209	0.0228	0.1150	0.0043	0.7898	0.0308	113.438	454.603
Model 7	0.7314	0.0333	0.1149	0.0044	0.7899	0.0309	117.438	470.788

**Table 5:** Results of RSF turnover prediction models with different models.

Model	C-Index	Up	IBS	Up	mean_AUC	Up
Baseline	0.7232	/	0.1138	/	0.7803	/
Model 1	0.7246	0.0014	0.1122	0.0016	0.7830	0.0027
Model 2	0.7229	−0.0003	0.1160	−0.0022	0.7833	0.0030
Model 3	0.7514	0.0282	0.1136	0.0002	0.8048	0.0245
Model 4	0.7283	0.0051	0.1157	−0.0019	0.7901	0.0098
Model 5	0.7514	0.0282	0.1121	0.0017	0.8071	0.0268
Model 6	0.7476	0.0244	0.1152	−0.0014	0.8056	0.0253
Model 7	0.7462	0.0230	0.1156	−0.0018	0.8049	0.0246

**Table 6:** Results of the tuned RSF turnover prediction model.

Model	C-Index	Up	IBS	Up	mean_AUC	Up
Baseline	0.7232	/	0.1138	/	0.7803	/
Model5	0.7514	0.0282	0.1121	0.0017	0.8071	0.0268
Best	0.7570	0.0338	0.1131	0.0007	0.8146	0.0343

## Data Availability

The data presented in this study are available on request from the corresponding author. The data are not publicly available due to privacy and ethical restrictions.

## References

[B1-behavsci-16-00174] Cai X., Shang J., Jin Z., Liu F., Qiang B., Xie W., Zhao L. (2020). DBGE: Employee turnover prediction based on dynamic bipartite graph embedding. IEEE Access.

[B2-behavsci-16-00174] Chang J. Y., Choi J. N., Kim M. U. (2008). Turnover of highly educated RD professionals: The role of pre-entry cognitive style, work values and career orientation. Journal of Occupational and Organizational Psychology.

[B3-behavsci-16-00174] Cho Y. J., Lewis G. B. (2012). Turnover intention and turnover behavior: Implications for retaining federal employees. Review of Public Personnel Administration.

[B4-behavsci-16-00174] Cohen G., Blake R. S., Goodman D. (2016). Does turnover intention matter? Evaluating the usefulness of turnover intention rate as a predictor of actual turnover rate. Review of Public Personnel Administration.

[B5-behavsci-16-00174] De Jesus A. C. C., Júnior M. E. G. D., Brandão W. C. (2018). Exploiting Linkedin to predict employee resignation likelihood. 33rd Annual ACM Symposium on Applied Computing.

[B6-behavsci-16-00174] Egelman S., Bonneau J., Chiasson S., Dittrich D., Schechter S., Blyth J., Dietrich S., Camp L. J. (2012). It’s not stealing if you need it: A panel on the ethics of performing research using public data of illicit origin. Financial cryptography and data security.

[B7-behavsci-16-00174] Fang F., Zhou Y., Ying S., Li Z. (2023). A study of the ping an health app based on user reviews with sentiment analysis. International Journal of Environmental Research and Public Health.

[B8-behavsci-16-00174] Fu R., Qin B., Liu T. (2013). Exploiting multiple sources for open-domain hypernym discovery. 2013 Conference on Empirical Methods in Natural Language Processing.

[B9-behavsci-16-00174] Ghosh P., Satyawadi R., Prasad Joshi J., Shadman M. (2013). Who stays with you? factors predicting employees’ intention to stay. International Journal of Organizational Analysis.

[B10-behavsci-16-00174] Goldberg D. M., Zaman N. (2018). Text analytics for employee dissatisfaction in human resources management. Twenty-fourth Americas Conference on Information Systems.

[B11-behavsci-16-00174] Guo F., Gallagher C. M., Sun T., Tavoosi S., Min H. (2024). Smarter people analytics with organizational text data: Demonstrations using classic and advanced NLP models. Human Resource Management Journal.

[B12-behavsci-16-00174] Gupta S., Sharma R. R. K. (2022). Types of HR analytics used for the prediction of employee turnover in different strategic firms with the use of enterprise social media. 5th International Conference on Industrial Engineering and Operations Management.

[B13-behavsci-16-00174] Hao L., Kim J., Kwon S., Ha I. D. (2021). Deep learning-based survival analysis for high-dimensional survival data. Mathematics.

[B14-behavsci-16-00174] Ishwaran H., Lauer M. S., Blackstone E. H., Lu M., Kogalur U. B. (2021). Randomforestsrc: Random survival forests vignette.

[B15-behavsci-16-00174] Jain D. (2017). Evaluation of employee attrition by effective feature selection using hybrid model of ensemble methods. Master’s thesis.

[B16-behavsci-16-00174] Jayaratne M., Jayatilleke B. (2022). Predicting job-hopping motive candidates using answers to open-ended interview questions. Journal of Computational Social Science.

[B17-behavsci-16-00174] Jin Z., Shang J., Zhu Q., Ling C., Xie W., Qiang B. (2020). RSF: Employee turnover prediction based on random forests and survival analysis. Web information systems engineering–WISE 2020: 21st international conference, Amsterdam, The Netherlands, 20–24 October 2020.

[B18-behavsci-16-00174] Juvitayapun T. (2021). Employee turnover prediction: The impact of employee event features on interpretable machine learning methods. 2021 13th International Conference on Knowledge and Smart Technology (KST).

[B19-behavsci-16-00174] Kammeyer-Mueller J. D., Wanberg C. R., Glomb T. M., Ahlburg D. (2005). The role of temporal shifts in turnover processes: It’s about time. Journal of Applied Psychology.

[B20-behavsci-16-00174] Korytkowski M., Nowak J., Scherer R., Zbieg A., Żak B., Relikowska G., Mader P. (2023). Employee turnover prediction from email communication analysis. Artificial intelligence and soft computing.

[B21-behavsci-16-00174] Li H., Ge Y., Zhu H., Xiong H., Zhao H. (2017). Prospecting the career development of talents: A survival analysis perspective. 23rd ACM SIGKDD International Conference on Knowledge Discovery and Data Mining.

[B22-behavsci-16-00174] Lloyd K. J., Boer D., Keller J. W., Voelpel S. (2015). Is my boss really listening to me? the impact of perceived supervisor listening on emotional exhaustion, turnover intention, and organizational citizenship behavior. Journal of Business Ethics.

[B23-behavsci-16-00174] Maimai, Yiguan (2018). Discovery and decryption of workplace groups in the rising digital workplace economy—Special analysis on user profiles of chinese workplace groups [Industry report]. Retrieved from Maimai & Yiguan.

[B24-behavsci-16-00174] Min H., Yang B., Allen D. G., Grandey A. A., Liu M. (2024). Wisdom from the crowd: Can recommender systems predict employee turnover and its destinations?. Personnel Psychology.

[B25-behavsci-16-00174] Mohiuddin K., Alam M. A., Alam M. M., Welke P., Martin M., Lehmann J., Vahdati S. (2023). Retention is all you need. 32nd ACM International Conference on Information and Knowledge Management.

[B26-behavsci-16-00174] Mulla Z. R., Kelkar K., Agarwal M., Singh S. (2023). Engineers’ voluntary turnover: Application of survival analysis. Indian Journal of Industrial Relations.

[B27-behavsci-16-00174] Ouyang C., Ma Z., Ma Z., Su J. (2023). Research on employee voice intention: Conceptualization, scale development, and validation among enterprises in China. Psychology Research and Behavior Management.

[B28-behavsci-16-00174] Priambodo B., Jumaryadi Y., Rahayu S., Ani N., Ratnasari A., Salamah U., Putra Z. P., Otong M. (2022). Predicting employee turnover in it industries using correlation and chi-square visualization. International Journal of Advanced Computer Science and Applications.

[B29-behavsci-16-00174] Qiao G., Bin W., Bai W., Baoli Z. (2019). Competency analysis in human resources using text classification based on deep neural network. 2019 IEEE Fourth International Conference on Data Science in Cyberspace (DSC).

[B30-behavsci-16-00174] Sajjadiani S., Sojourner A. J., Kammeyer-Mueller J. D., Mykerezi E. (2019). Using machine learning to translate applicant work history into predictors of performance and turnover. Journal of Applied Psychology.

[B31-behavsci-16-00174] Saradhi V. V., Palshikar G. K. (2011). Employee churn prediction. Expert Systems with Applications.

[B32-behavsci-16-00174] Sharada K. R., Arya A., S R., Kumar H., G A. (2012). A text analysis based seamless framework for predicting human personality traits from social networking sites. International Journal of Information Technology and Computer Science.

[B33-behavsci-16-00174] Speer A. B., Perrotta J., Tenbrink A. P., Wegmeyer L. J., Delacruz A. Y., Bowker J. (2023). Turning words into numbers: Assessing work attitudes using natural language processing. Journal of Applied Psychology.

[B34-behavsci-16-00174] Van Dijck J. (2013). ‘You have one identity’: Performing the self on Facebook and LinkedIn. Media, Culture and Society.

[B35-behavsci-16-00174] Van Knippenberg D., van Dick R., Tavares S. (2007). Social identity and social exchange: Identification, support, and withdrawal from the job. Journal of Applied Social Psychology.

[B36-behavsci-16-00174] Yuan S., Kroon B., Kramer A. (2024). Building prediction models with grouped data: A case study on the prediction of turnover intention. Human Resource Management Journal.

[B37-behavsci-16-00174] Zheng Y., Long Y., Fan H. (2022). Identifying labor market competitors with machine learning based on Maimai platform. Applied Artificial Intelligence.

[B38-behavsci-16-00174] Zhu Q., Shang J., Cai X., Jiang L., Liu F., Qiang B. (2019). COXRF: Employee turnover prediction based on survival analysis. 2019 IEEE SmartWorld, Ubiquitous Intelligence Computing, Advanced Trusted Computing, Scalable Computing Communications, Cloud Big Data Computing, Internet of People and Smart City Innovation (SmartWorld/SCALCOM/UIC/ATC/CBDCom/IOP/SCI).

